# Study of the Extraction Process and *In Vivo* Inhibitory Effect of Ganoderma Triterpenes in Oral Mucosa Cancer

**DOI:** 10.3390/molecules16075315

**Published:** 2011-06-24

**Authors:** Yang Gao, Ruhui Zhang, Juan Zhang, Shang Gao, Wenxin Gao, Haifeng Zhang, Haotian Wang, Bing Han

**Affiliations:** 1 Stomatology Hospital, Jilin University, Changchun 130021, Jilin, China; 2 School of Pharmacy, Jilin University, Changchun 130021, Jilin, China

**Keywords:** extraction process, ganoderma triterpenes, oral cancer, immunohistochemistry

## Abstract

The aim of the reported study was to optimize the extraction process for ganoderma triterpenes and to investigate the *in vivo* inhibitory effect of ganoderma triterpenes on the genesis and progression of oral cancer. Single-factor and orthogonal methods were used to investigate the effects of extraction solvent, solvent amount, extraction time, extraction temperature, and number of extractions, on the extraction rate for ganoderma triterpenes. A golden hamster model with cheek pouch dynamic canceration was established to receive oral treatment of ganoderma triterpenes water solution. Animals were continuously monitored, oral tissue samples were collected for histopathologic examination, and changes in the expression of VEGF (vascular endothelial growth factor) and Caspase-3 were detected by immunohistochemical methods. Optimization of the experimental conditions allowed the identification of the optimal extraction conditions: 90% ethanol as the extraction solvent, a solvent amount by the liquid-material ratio of 35 mL/g, extraction time of 2 h and extraction temperature of 80 °C. Under these conditions, the average extraction rate of ganoderma triterpenes was 1.09%. Tests in golden hamsters showed that compared with the model group during the same period, animals in the treatment group had better conditions, constantly larger number of normal cases shown by histopathologic results (P < 0.01), and consistently smaller numbers of cases with paraplasm (P < 0.05). Immunohistochemical results showed that compared with the model group, the treatment group had significantly lower (P < 0.05) rates of positive VEGF expression in the normal state, simple epithelial hyperplasia, epithelial dysplasia or squamous cell carcinoma disease stages. Caspase-3 expression showed a tendency toward a gradual increase with the worsening of disease severity in each group. Compared with the model group, the treatment group had significantly lower (P < 0.05) rates of positive Caspase-3 in the normal state, simple epithelial hyperplasia, epithelial dysplasia or squamous cell carcinoma disease grades. Using the optimized extraction process, ganoderma triterpenes could be extracted with high efficiency, and the results of animal tests showed inhibitory effects of ganoderma triterpenes on oral mucosa cancer.

## 1. Introduction

Ganoderma triterpene is the main chemical and active component of ganoderma. Research has shown that triterpene compounds have significant physical activity, especially including antitumor actions [1,2]. The extraction rate of ganoderma triterpenes is low due to its low content and the hard, dense structure of its fruiting body [3]. The extraction and separation of triterpene components in ganoderma have been reported in several studies: Ma Lin *et al.* [4] analyzed four medicinal triterpene components (ganosporeic acid A, lucidenic acid A, ganodenic acid B, ganodenic acid C) in the ganoderma fruiting body of different origins by HPLC; Ma Jian-Yan [5] obtained three types of triterpenes through separation and purification from the ganoderma fruiting body by chromatographic extraction, and the structures of the separated products were analyzed using UV-Vis, ESI and ^1^H-NMR. The methods for extraction of ganoderma triterpenes mainly include: (1) Extraction with methanol or ethanol as the solvent, followed by direct separation of the extracts; (2) Extraction by methanol or ethanol, followed by separation of the total acid portion by alkali treatment and separation of ganoderma triterpene; and (3) Extraction of the total acid portion by ether, followed by diazomethane methylation and then separation. Extraction of ganoderma triterpene using ethanol is the easiest approach to maintain the activity of the extracts and to scale-up its production. In this article, single-factor and orthogonal methods were used to investigate the effects of extraction solvent, solvent amount, temperature, and time on the extraction rate of ganoderma triterpenes, so as to obtain the optimal process parameters and provide a reference and evidence for the deep processing of ganoderma.

Ganoderma triterpenes have inhibitory effects on lung cancer, liver cancer, and colorectal cancer. Among the studies on cell lines, Tang Qing-Jiu *et al.* [6] extracted and separated the neutral components of ganoderma triterpene for *in vitro* oncostatic tests on several cell lines including SW620 cells (human intestinal cancer cells) and K562 cells (human lymph cancer cells). Neutral triterpenes in ganoderma were found to have an inhibitory effect on the proliferation of various tumor cells and induced apoptosis of intestinal cancer SW620 cells. Using an MTT method, Zheng Lin *et al.* [7] have observed that extracted triterpene components can inhibit tumor cell growth and that ganoderma triterpenes has inhibitory effects on various cancer cell lines, especially on skin and liver cancers. There has also been a report [8] that ganoderma triterpenes can significantly inhibit the proliferation of a highly metastatic lung cancer cell line (95-D) by inducing apoptosis and blocking the cell cycle. Ganoderma triterpenes can inhibit topoismerase IIa, thus inhibiting the synthesis of DNA and inducing apoptosis in HuH-7 human liver cancer cells. In addition to the antitumor effect, ganoderma triterpenes has also shown hypolipemic action, as it contains ganoderol A, ganoderol B and ganoderal A, which have the biologic effect of inhibiting cholesterol synthesis in human liver cell lines. Some other compounds in ganoderma triterpenes have an inhibitory effect on the inflammation mediators released from neutrophils and other cells [9]. Furthermore, ganoderma triterpenes has liver-protective, antibacterial, antiviral and antifungal pharmacological actions [10,11]. Therefore, ganoderma triterpenes is a highly promising candidate for pharmacological research and clinical application. However, to date there have been no studies examining its effect on oral cancer, nor have there been reports on its use as an adjuvant therapy for OSCC (oral squamous cell carcinoma), one of the most common malignant tumors. Oral leukoplakia, the most common pre-cancerous change, leads to a canceration rate of 6.88% at the average age of 59.13 years, with an average of 4.2 years since leukoplakia. Because the pathological process from normal oral mucosa to OLK and to OSCC is relatively long, treatment with natural or synthetic chemical drugs for pre-cancerous oral changes can block or even reverse the genesis and progression of cancer. These chemicals may represent one effective way to lower the canceration rate.

For this study, a golden hamster model was used in which cheek pouch dynamic canceration was induced by DMBA (7,12-dimethylbenz(a)anthracene). Animals in the treatment group were observed over 12 weeks for pathologic changes versus the model group. Changes in the expression of VEGF and Caspase-3 during the canceration process were also detected to investigate the inhibitory effect of ganoderma triterpenes on the genesis and progression of oral cancer.

## 2. Test Results

### 2.1. Preparation of Standard Curve

The standard curve was prepared as directed under “5.1”, and the absorption values were recorded. By linear regression with absorption (Y) as the vertical ordinate and the amount of the standard (X, mg) as the horizontal ordinate, the following regression equation was obtained:

Y = 5.78x − 0.0022 correlation coefficient r = 0. 9994.

**Figure 1:**
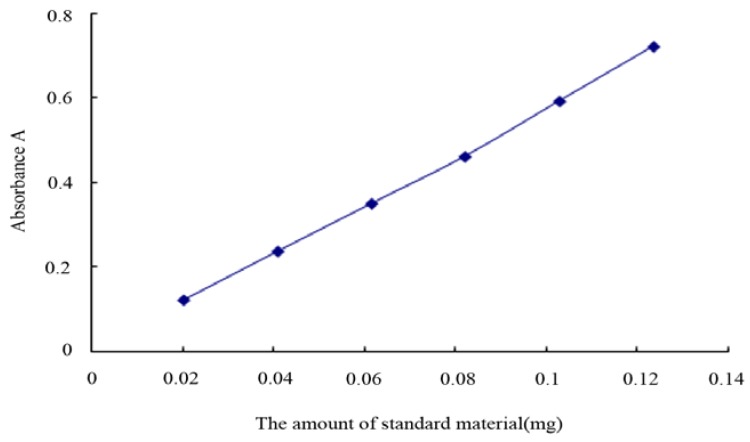
Ursolic acid standard curve.

The results indicate that the ursolic acid standard exhibited a good linear relationship with absorption values in the mass range of 0.0206 ~ 0.1236 mg, in which the results were accurate. The standard curve is presented in Figure 1.

### 2.2. Results Analysis of Single-Factor Test

#### 2.2.1. Result of the influence of extraction solvent on the extraction rate

**Figure 2:**
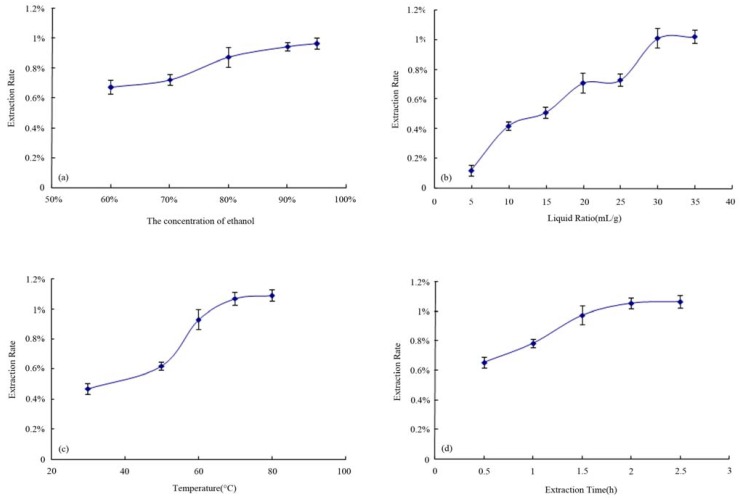
Results of single-factor tests.

See Figure 2(a) for the result. The extraction rate increased with the increase of ethanol concentration up to 80%, followed by slowed increases in the extraction rate. The three levels of extraction solvent for the orthogonal test were finally determined to be 80% ethanol, 90% ethanol and 95% ethanol.

#### 2.2.2. Result of the influence of liquid-material ratio on the extraction rate

See Figure 2(b) for the result. When the liquid-material ratio was 10 mL/g, the solvent failed to fully cover the ganoderma material, resulting in a low extraction rate; the extraction rate increased with the increase in the liquid-material ratio. All ratios above 20 mL/g resulted in high extraction rates, while the ratio of 30 mL/g produced an extraction rate that was similar to that produced by 35 mL/g. The three levels of liquid-material ratio for the orthogonal test were finally determined to be 20 mL/g, 30 mL/g and 35 mL/g.

#### 2.2.3. Result of the influence of extraction temperature on the extraction rate

See Figure 2(c) for the result. The extraction rate for ganoderma triterpenes increased as temperatures increased below 80 °C, but increased to a small extent with temperatures between 80 °C to 100 °C. The three levels of extraction temperature for the orthogonal test were finally determined to be 80 °C, 90 °C and 100 °C.

#### 2.2.4. Result of the influence of extraction time on the extraction rate

See Figure 2(d) for the result. The extraction rate for ganoderma triterpenes increased with the prolongation of the extraction time up to 1 h, but increased to a small extent after 1 h. The three levels of extraction time for the orthogonal test were finally determined to be 1.5 h, 2 h and 2.5 h.

### 2.3. Analysis of the Orthogonal Test Results

#### 2.3.1. Determination of the optimal process

Total ganoderma triterpenes were extracted in the orthogonal test based on the levels of each factor determined in the single-factor test. The factor levels are presented in Table 1, and the results of the orthogonal test are presented in Table 2; see Table 3 for the ANOVA results.

Table 2 and Table 3 show that given the R of only 0.057, ethanol concentration had little effect on the extraction rate, which was slightly higher with an ethanol concentration of 90%. The extraction rate was the highest with a liquid-material ratio of 35 mL/g and very low with a liquid-material ratio of 20 mL/g. The extraction rate was the highest with an extraction time of 2 h or 2.5 h, with little difference between these times, while the extraction rate was low with an extraction time of 1.5 h. The extraction rate increased with an increase in the extraction temperature and reached a peak at 80 °C. The solvent volume was a significant influencing factor (F = 29.20, P < 0.05), while ethanol concentration, extraction time and extraction temperature had little influence. In view of these findings, and considering the economic factor, the optimal condition for ganoderma triterpene extraction was determined to be A2B3C2D3, that is, 90% ethanol, solvent volume in the liquid-material ratio of 35 mL/g, extraction time of 2 h and extraction temperature of 80 °C.

#### 2.3.2. Validation test

To validate the technological conditions, we took three aliquots of the same batch of material for extraction under the condition of A2B3C2D3 as directed under “5.3”. The extraction rate of ganoderma triterpenes was determined to be 1.09%.

### 2.4. Results of Daily Observation of Test Animals

At Week 3, the golden hamsters in both groups displayed good mental states and no abnormalities; body weights in both groups showed a tendency toward raising and the mucosa of hamsters was smooth and ruddy with good flexibility. At Week 6, the animals in the model group had slightly smooth hair and a fair mental state. The mucosa in these animals was thickening and becoming rough with decreased flexibility. The treatment group had a significantly larger body weight than Group B, which showed smooth hair, good mental state, no notable mucosa thickening, mostly smooth and ruddy mucosa with good flexibility, and visible submucous vessels. At Week 9, the model group had slightly rough hair, average mental state, significant mucosa thickening with white plague or papilate convex, neoplasms tending to bleed upon probing in some animals, and mucosa with increased fragility in operational separation. The treatment group had somewhat decreased body weight (which was still higher than in the model group), slightly smooth hair, good mental state, and thickening and coarsening mucosa with occasional and small neoplasms. At Week 12, the model group had rough hair, poor mental state, poor appetite and marked emaciation, indicating a consumptive condition; most mucosa had cauliflower neoplasms that had a red appearance, tended to bleed upon touch, and were quickly growing toward the oral cavity, causing cheek pouch eversion and difficulty in opening the mouth. The mucosa in these animals was extremely fragile upon operational separation. The model and treatment groups showed no significant difference in body weight. The treatment group had slightly rough hair, average mental state, notably thickening mucosa with white plaque on the surface, and a small number of papillary neoplasms in isolated cases.

### 2.5. Results of Pathologic Observation

**Figure 3:**
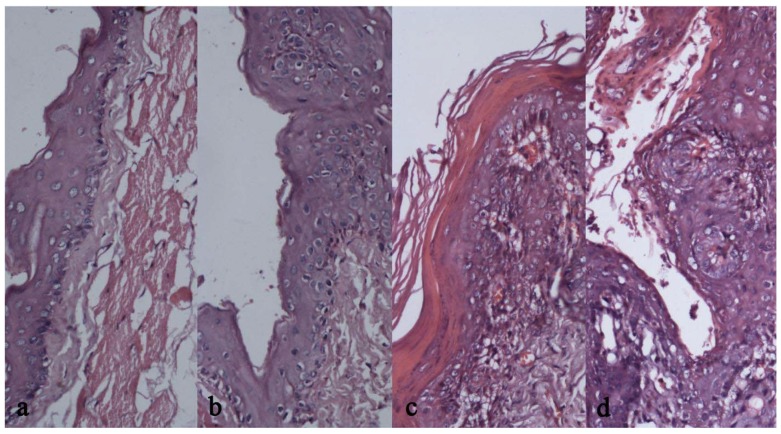
(**a**). Tissue sections at 9 weeks in the Treatment Group; (**b**). Tissue sections at 9 weeks in the Model Group; (**c**). Tissue sections at 12 weeks in the Treatment Group; (**d**). Tissue sections at 12 weeks in the Model Group.

The histopathologic observations are separately presented in Table 4 and Table 5. It can be seen that treatment group had constantly larger number of normal cases than model group, P < 0.01 by the χ2 test. The treatment group had consistently smaller numbers of paraplasm than the model group, P < 0.05 by the χ2 test. Table 6 presents the incidences of different histological states, which had significant differences between the two groups by the rank-sum test (P < 0.01). Histopathologic changes were observed microscopically with HE staining (Figure 3). In the blank group, keratinized stratified squamous epithelium with regular cell arrangement, no rete pegs or no obvious rete pegs were observed, a prickle cell layer that was thinner than in normal human mucosa and the basal layer cells were obvious. Comparison of epithelial dysplasia revealed that at Week 3, compared with the treatment group, the model group had irregular epithelial layers, darker nuclei with larger nucleoli with the majority of basal layer cells losing polarity, and increased mitotic figures. At Week 6, one animal in the model group developed serious non-typical hyperplasia involving most of the epithelium that was hardly reversible. At Week 9, one animal in the control group was assessed as OSCC based on obvious cell heteromorphism, epitheliosis intruding the connective tissues in blocks or streaks, and part of the epithelium exhibiting obvious horny pearls, increased mitotic figures, and polymorphism of cells and nuclei. After Week 12, squamous cell carcinoma appeared in both model and treatment groups, but with significantly fewer animals with squamous cell carcinoma in the treatment group, and more serious mitotic phase increases in the model group. Among them, 5 animals in the treatment group had keratized epithelium, the majority had hyperkeratosis and the minority had dyskeratosis, acanthosis, basal membrane remaining regular, and infiltration of lymphocytes and plasmacytes under the proper layer. All of these animals showed general inflammatory reactions, while paraplasm or squamous cell carcinoma that was more serious than in these animals was observed in the model group.

### 2.6. Results of Immunohistochemical Staining

#### 2.6.1. Results of VEGF immunohistochemical stsaining

**Figure 4:**
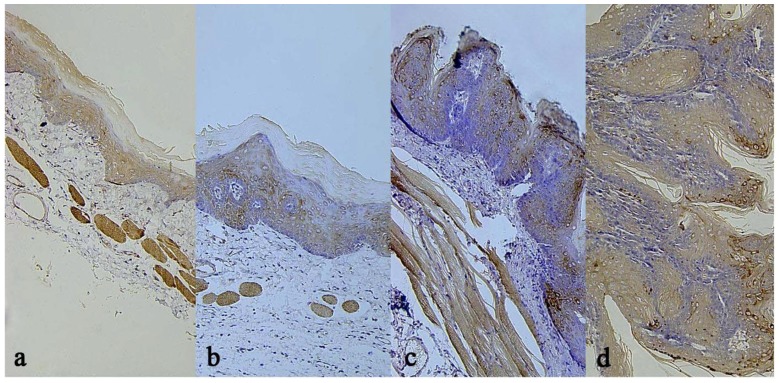
Tissue sections at 9 weeks corresponds VEGF immunostaining. (**a**). Dysplasia epithelium in the Treatment Group; (**b**). Dysplasia epithelium in the Model Group; (**c**). Squamous cell carcinoma in the Treatment Group; (**d**). Squamous cell carcinoma epithelium in the Model Group.

The immunohistochemical results are presented in Table 6. Compared with the model group, the treatment group had lower positive expression rates of VEGF (P < 0.05) in the normal, inflammation, paraplasm or squamous cell carcinoma states. These data indicate that compared with the model group, the treatment group had fewer and lower grade blood vessel hyperplasia at each disease stage (Figure 4).

#### 2.6.2. Results of Caspase-3 immunohistochemical staining

**Figure 5:**
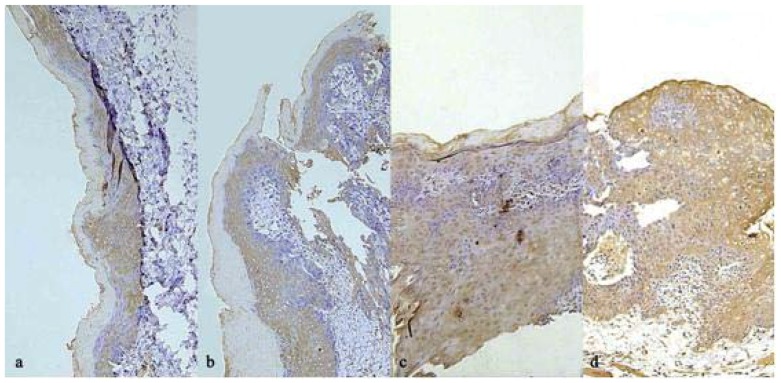
Tissue sections at 9 weeks corresponds caspase-3 immunostaining. (**a**). Dysplasia epithelium in the Treatment Group; (**b**). Dysplasia epithelium in the Model Group; (**c**). Squamous cell carcinoma the Treatment Group; (**d**). Squamous cell carcinoma epithelium in the Model Group.

The immunohistochemical results are presented in Table 7. Compared with the model group, the treatment group had lower positive expression rates of Caspase-3 (P < 0.05) in the normal, inflammation, paraplasm or squamous cell carcinoma states. These data indicate that compared with the model group, the treatment group had fewer and lower grade epithelial lesions at each disease stage (Figure 5).

## 3. Discussion

The results showed that among the extraction conditions for ganoderma triterpene, solvent amount and temperature were two major influences on the extraction rate, while extraction time over 1 hour had little effect on extraction rate. Thus, a better method to reduce the energy consumption during the extraction process by shortening the extraction time is yet to be developed. The ethanol concentration may also be reduced as appropriate to reduce the residual ethanol in the ganoderma triterpenes, since ethanol concentrations over 80% had little influence on the extraction rate. The results of this study showed that the process conditions of 90% ethanol as the extraction solvent, a solvent amount by the liquid-material ratio of 35 mL/g, extraction time of 2 h and extraction temperature of 80 °C resulted in good stability and in a high extraction rate of ganoderma triterpene and therefore can be used for the large-scale extraction of ganoderma triterpene from ganoderma.

OSCC is the most common malignancy in the oromaxillo-facial region, accounting for over 80% of the oromaxillo-facial malignancies and 3%–5% of overall malignancies. Histologic examination has shown that oral squamous epithelial dysplasia can cause the formation oral leukoplakia that clinically leads to a considerable canceration rate. To investigate whether ganoderma triterpenes have an inhibitory effect on oral mucosa squamous cell carcinoma, and whether the inhibitory effect, if present, is phased and consistent, a golden hamster model with DMBA-induced cheek pouch dynamic canceration was used [12]. Test animals orally treated with ganoderma triterpenes and those in the model group not treated with this drug were observed systematically at Weeks 6, 9 and 12 for daily living state and histopathologic changes. Observations of the daily living state and results of HE staining of oral tissues showed that compared with the model group, the golden hamsters in the treatment group had significantly reduced lesions, indicating the inhibitory effect of ganoderma triterpenes on oral cancer. Furthermore, the expression of VEGF and caspase-3 were detected by immunohistochemical methods in the course from the normal state to simple epithelial hyperplasia to epithelial dysplasia and to squamous cell carcinoma for both groups.

VEGF is an exocrine protein that produces its effect by both paracrine and autocrine pathways. This protein is a highly specific vascular endothelial cell mitogen that plays the vital role of promoting endothelial cell division and proliferation and enhancing capillary permeability [13]. The increase in capillary permeability causes extravasation of plasma protein and fibrinogen, resulting in changes in the cell matrix. These effects facilitate angiopoiesis and the formation of a new matrix, making possible the growth, infiltration and metastasis of tumor. The rate of positive VEGF expression is closely related to the infiltration and metastasis s or occasionally in isolated vascular endothelial cells of the subepithelial connective tissues. In OSCC, positive reactions, mostly strong, were frequently observed in the cytoplasm of tumor cells. In tumor interstitial substance, positive reactions increased in vascular endothelia cells and were also observed in some inflammatory cells. The intensity of VEGF expression increased with the development of mucosa cancer in both groups. Normal mucosa staining was negative in both groups. Throughout the canceration process, the intensity of VEGF expression increased with the development of mucosa cancer in both groups, indicating that angiogenesis has a vital role in tumor growth and metastasis. Numerous studies have shown that VEGF is the most effective growth factor for tumor angiogenesis, which, if uncontrolled, plays a dominating role in tumor growth and metastasis. Immunohistochemical staining results in the model group showed that angiogenesis was present in both pre-cancerous affection and canceration stages and increased significantly with the worsening in the malignancy grade. In each stage, the VEGF positive expression rate was lower in the treatment group than in the model group, indicating that ganoderma triterpenes has an inhibitory effect on blood vessel hyperplasia.

The cysteinyl aspartate specific proteinase (caspase) family is a group of cysteine-containing proteases that play an important role in apoptosis by inducing efficient and specific proteolysis in agonal cells. As the executors of apoptosis, they determine the morphological and biochemical changes leading to apoptosis [14]. Caspase 3, an important member of this family, is a key executor of apoptosis [15]. Not only is Caspase-3 tissue-specific in its expression in tumor, it also plays different roles in the progression of different tumor tissues.

Caspase-3 positive staining (brownish yellow or dark brown) was primarily distributed in the cytoplasm and occasionally in nuclei. Caspase-3 was not or was weakly expressed in normal tissues by cytoplasm coloration or occasional nuclei coloration in the lower prickle cell layer or in very few superficial cells. The expression of caspase-3 increased with the worsening of epithelial lesions. In the tissues of severe epithelial dysplasia, positive cells pervaded all epithelial layers. Caspase-3 was widely expressed in squamous cell cancerous tissues. Compared with the model group, the treatment group had lower positive rates in the normal, simple epithelial hyperplasia, epithelial dysplasia or squamous cell carcinoma disease stages.

This study demonstrates that in the course from the normal state to simple epithelial hyperplasia to epithelial dysplasia and to squamous cell carcinoma, caspase-3 expression showed a tendency toward a gradual increase in both groups, indicating that the caspase-3 expression level may be consistent with the progression form pre-oral cancerous changes to squamous cell carcinoma. Therefore, caspase-3 activation may be a biomarker for active pre-cancerous changes.

Caspase-3 has different effects in regulating the apoptosis of tumor cells of different histological origins, and varies in the expression in different tumor tissues. In most types of carcinoma, its expression is inhibited with growing histological malignancy in the tumor development, however it may result in different outcomes in some carcinomas, as in the process from normal esophageal epithelium to atypically hyperplastic epithelium then to esophageal squamous cell carcinoma in esophageal carcinoma, there the initial up-regulation of caspase-3 expression is followed by a down-regulation, leading to overexpression of caspase-3 in esophageal carcinoma, while in malignant lymphoma and medulloblastoma, the expression increases with the evolving malignancy. It is assumed that increased caspase expression maintains the cell number at a stable level by promoting apoptosis and thus inhibiting cell proliferation, but due to the proliferation overwhelming the apoptosis defense the absolute cell apoptosis rate is low, leading to constant accumulation of dysplasia cells. In the present study, as the squamous cell carcinoma we induced had low malignancy with high differentiation, it was not possible to verify the relationship between changes in caspase- 3 expression and the increased squamous carninoma level, necessitating further studies with extended induction time. We believe that positive caspase-3 expression in oral squamous cell carcinoma indicates higher differentiation of cancer cells, lower blocking on cell apoptosis regulation mechanism and lower proliferation of cancer cells, indicating better prognosis. The slightly lower positive rate observed in Treatment group than in Model group may have been the result of lower malignancy, less active cell proliferation and weaker apoptosis in Treatment group. This also suggests that Ganoderma triterpene inhibits the generation and development of oral squamous carcinoma. In our study, no increased caspase-3 expression was noted in the Treatment group as compared to the Model group. We assume that in oral squamous cell carcinoma, instead of inducing cell apoptosis through caspase-3 activation, ganoderma triterpenes may have exerted direct cytotoxic effect on tumor cells.

Because the progression from normal oral mucosa to squamous cell carcinoma involves gradual and prolonged changes, it is clinically important to inhibit the genesis and development of cancer by effective drugs to lower the incidence of squamous cell carcinoma. This effort has drawn wide attention from the research community. Many Chinese medicines are currently applied as adjuvant therapies for cancers and have been clinically proven to provide a wide variety of options for cancer inhibition. This study demonstrates that ganoderma triterpenes have inhibitory effects on oral mucosa squamous cell carcinoma and provide a feasible option for clinical treatment. 

## 4. Materials

### 4.1. Test Drug and Reagents

The following reagents were used in this study: Ganoderma: Wild strains of *Ganoderma lucidum* have been used in this study. They were collected in Changbai Mountains. Due to the year-long cold climate with short frost-free period and large day-night temperature differences, *Ganoderma lucidum* from Changbai Mountains has long growth cycle and low yield, but provides higher amounts of nutritional and pharmaceutical ingredients as compared to the artificial cultivars, with more solid and thicker texture as well as better luster. The fruit body is an non-toxic, annual growth with irregular shape and a particularly bitter taste, ursolic acid control (China Institute for the Control of Pharmaceutical and Biological Products, purity >98%), dimethyl benzanthracene, (9,10-dimethyl-1, 2-benzanthracene, DMBA, Sigma), analytically pure acetone solution (Changchun Medical Supplies Co., Ltd.), SP immunohistochemistry kit (Shanghai Bluegene Biotech Co., Ltd.), VEGF polyclonal antibody (Shanghai Jingtian Biotech Co., Ltd), Caspase-3 polyclonal antibody (Wuhan Boster Biological Technology Co., Ltd.), condensed DAB kit (Tianjin Bomeike Biotechnology Co., Ltd.) and analytical pure grades of alcohol, glacial acetic acid, perchloric acid and citroellal. Five percent glacial acetic acid- vanillin solution was prepared by dissolving 0.5 g of vanillin in 10 mL of glacial acetic acid.

### 4.2. Instruments

The following instruments were used: UV-5600 ultraviolet-visible spectrophotometer (Shanghai Metash Instruments Co., Ltd.), R206D rotatory evaporator (Shanghai Senco Technology Co., Ltd.), HH-2 digital display thermostatic waterbath (Jintan Union Instruments Research Institute), and AL104 electronic balance (Mettler-Toledo).

## 5. Methods 

### 5.1. Preparation of Standard Curve

The routine determination of total triterpenes in ganoderma uses ultraviolet-visible spectrophotometry with a triterpene compound such as ursolic acid as the control and 5% glacial acetic acid- vanillin solution and perchloric acid as the developer [16]. The protocol for preparation of the standard curve is as follows: ursolic acid standard (10.3 mg) is transferred into a 10 mL volumetric flask, dissolved and made up to volume with ethyl acetate to make a 1.03 mg/mL perchloric acid stock solution. The stock solution (1 mL) is transferred into a 10 mL volumetric flask, made up to volume with ethyl acetate and shake well to make a 0.103 mg/mL perchloric acid standard solution. Separately perchloric acid standard solution (0.20, 0.40, 0.60, 0.80, 1.00 and 1.20 mL) is transferred into tubes with a stopper, evaporated to dryness on a 100 °C waterbath, 5% glacial acetic acid- vanillin solution (0.40 mL) and perchloric acid (1.00 mL) are added, and the mixture heated for 25 min in a 60 °C waterbath and then transferred to an ice water bath. Glacial acetic acid (5.0 mL) is added and the mixture shaken well before placing the tubes at room temperature. Fifteen minutes later, the absorption at 548.2 nm was determined on the ultraviolet-visible spectrophotometer, with the reagent as the blank control. The standard curve was prepared as per the determination results [17,18].

### 5.2. Treatment and Assay of Samples

The volume of the triple extraction mixture of each sample was accurately measured, 1/100 of the mixture was pipetted as for the test sample, evaporated to dryness on a 100 °C waterbath, 5% glacial acetic acid- vanillin solution (0.40 mL) and perchloric acid (1.00 mL) were added, the mixture was heated for 25 min in a 60 °C waterbath and then transferred to an ice water bath. Glacial acetic acid (5.0 mL) was added and the mixture was shaken well before placing at room temperature. Fifteen minutes later, the absorption of the sample solution was determined at 548.2 nm on the ultraviolet-visible spectrophotometer.

### 5.3. Single-Factor Test

#### 5.3.1. Extraction of ganoderma triterpenes

Granulated ganoderma fruiting body was placed in a round bottomed flask for the single-factor test of the extraction process, where the effects of the factors including extraction solvent, solvent amount, extraction time, and extraction temperature, and so forth, on the extraction rate were investigated [4,19]. For this study, ethanol extraction was used for the preparation of the ganoderma triterpenes.

#### 5.3.2. Influence of extraction solvent on the extraction rate

One gram of each of the ganoderma fruiting body granules was placed in five 100 mL round bottomed flasks, where 60%, 70%, 80%, 90% and 95% ethanol, respectively, were added with a liquid-material ratio of 20 mL/g. Reflux extraction was repeated three times under 80 °C, for 2 h each time, and the three extracts were combined to determine the amount of ganoderma triterpenes and thus calculate the extraction rate.

#### 5.3.3. Influence of solvent amount on the extraction rate

The optimal solvent was determined based on the results of 5.3.2. One gram of each of the ganoderma fruiting body granules was placed in five 100 mL round bottomed flasks, where the extraction solvents of different volumes were added with the liquid-material ratios of 10, 15, 20, 30, and 35 mL/g. Reflux extraction was repeated three times under 80 °C, for 2 h each time, and the three extracts were combined to determine the amount of ganoderma triterpenes and thus calculate the extraction rate.

#### 5.3.4. Influence of extraction temperature on the extraction rate

The optimal solvent and solvent amount were determined based on the results of 5.3.2 and 5.3.3. One gram of each of ganoderma fruiting body granules was placed in five 100 mL round bottomed flasks, where the selected volume of the extraction solvent was added. Reflux extraction was repeated three times at 30, 50, 80, 90 and 100 °C, respectively, for 2 h each time. Then the three extracts were combined to determine the amount of ganoderma triterpenes and thus calculate the extraction rate. 

#### 5.3.5. Influence of extraction time on the extraction rate

The optimal solvent, solvent amount and extraction temperature were determined based on the results of 5.3.2, 5.3.3 and 5.3.4. One gram each of ganoderma fruiting body granules were placed in five 100 mL round bottomed flasks, where the selected volume of extraction solvent was added. Reflux extraction was repeated three times, for 0.5 min, 1 min, 1.5 h, 2 h and 2.5 h, respectively, each time. Then, the three extracts were combined to determine the amount of ganoderma triterpenes and thus calculate the extraction rate.

### 5.4. Orthogonal Test

One gram of ganoderma fruiting body granules was transferred to a 100 mL round bottomed flask, the extraction solvent was added, and reflux extraction was performed under a certain temperature. The reflux extracts were combined, the volume was accurately measured, and 1/100 of the extract was taken as the test sample. For this study, an L9 (34) orthogonal test was designed for the investigation of ganoderma triterpenes in the test samples based on the test factors of (A) ethanol concentration; (B) solvent-material ratio; (C) extraction time and (D) extraction temperature.

### 5.5. Selection and Grouping of Test Animals

Eighty-eight Syrian golden hamsters, half males and half females, 6–8 weeks of age, and with body weights of 100 ± 10 g were randomized to either the blank group with eight animals, the model group with 40 animals, or the treatment group with 40 animals. Female and male animals were separately caged under the conditions of simulated light-dark cycle, air ventilation, temperature of 25 ± 2 °C, humidity of 80 ± 5% and were given a complete pellet diet and city water. All animal protocols were approved by the Institutional Animal Care and Use Committee of Jilin University.

The chemical carcinogenic agent 0.5% DMBA in acetone solution was applied to the bilateral cheek pouches of animals on days 1, 3 and 5 of each week to prepare the models. After drying, the ganoderma triterpenes alcohol extract was dissolved in 0.5% sodium carboxymethyl cellulose to produce the ganoderma triterpenes liquid drug (10 g of crude drug/L). See Table 8 for group allocation and disposition of test animals.

### 5.6. Daily Observation of Test Animals

The activities of the golden hamsters were observed, the hair color and behavior were recorded, and the genesis and development of oral mucosa cancer were monitored.

### 5.7. Histologic Examination

Unilateral cheek pouch samples of 1.0 cm × 1.0 cm were taken from each hamster and subjected to conventional sectioning and HE staining. The histological severities of the lesions in each group were compared by grading diagnosis with double check based on the WHO Pre-Oral Cancerous Lesion Cooperation Center 12-item diagnosis criteria for epithelial dysplasia.

### 5.8. Immunohistochemical Staining

Cheek pouch specimens of the opposite side were taken from each hamster, from which two sections of affected cheek pouch tissue were made. Immunohistochemical staining was performed separately for VEGF and caspase-3.

#### 5.8.1. VEGF immunohistochemical staining

The immunohistochemical SP method was adopted as per the SP kit instructions. PBS buffer was used as the blank control in place of the antibody, and sections of known positive human vascular tumor were used as the VEGF staining positive control. The staining intensity was assessed by a semiquantitative method. Granular cytoplasm coloration of light to deep brown was considered to be VEGF positive staining. For each typical site chosen, 10 fields were randomly selected at high magnification (×400), and 100 cells were counted in each field to record the number of positive cells and calculate the mean percentage. The intensity was scored as one of the following grades: Grade 0 if positive cells <10%, Grade 1 if positive cells 10% ~ 50%, or Grade 2 if positive cells >50%. For analysis, Grade 0 was considered to be negative expression, while Grades 1 and 2 were considered to be positive expression.

#### 5.8.2. Caspase-3 immunohistochemical staining

The immunohistochemical SP method was adopted as per the SP kit instructions. PBS buffer was used as the blank control in place of the antibody, and sections of known positive breast cancer were used as the Caspase-3 staining positive control. The staining intensity was assessed by a semiquantitative method. Granular coloration of light to deep brown of the cytoplasm or nucleus was considered to be caspase-3 positive staining. For each typical site chosen, 10 fields were randomly selected at high magnification (×400), and 100 cells were counted for each field to record the number of positive cells and calculate the mean percentage. The intensity was scored as one of the following grades: Grade 0 if positive cells <25%, Grade 1 if positive cells 25% ~ 50%, or Grade 2 if positive cells >50%. For analysis, Grade 0 was considered to be negative expression, while Grades 1 and 2 were considered to be positive expression.

### 5.9. Statistical Treatment

A χ^2^ test was used for comparison of pathological results between samples. A Rank-sum test (the Wilcoxon Mann Whitney U-test) for grouped comparison between samples was primarily used for the immunohistochemical staining results. A two-sided α of 0.05 was adopted for assessment.

## 6. Conclusions

In this study, single-factor and orthogonal methods were used to investigate the factors influencing the extraction method that are most easily subject to process amplification, including the effects of extraction solvent, solvent amount, extraction time, and extraction temperature, on the extraction rate for ganoderma triterpenes. Considering the economic factor, the optimal condition for ganoderma triterpene extraction was determined to be 90% ethanol, a solvent volume in the liquid-material ratio of 35 mL/g, an extraction time of 2 h and an extraction temperature of 80 °C. A golden hamster model with cheek pouch dynamic canceration was established to receive oral treatment of a ganoderma triterpene water solution. Animal tissue samples were then collected for histologic examination, and changes in the expression of VEGF and caspase-3 in animals were detected by immunohistochemical methods. The study results prove the inhibitory effect of ganoderma triterpenes on oral mucosa cancer, providing a new paradigm for the treatment of OSCC.

## Figures and Tables

**Table 1:** Factor levels.

Factor level	A (%)	B (mL/g)	C (h)	D (°C)
1	80	20	1.5	60
2	90	30	2	70
3	95	35	2.5	80

**Table 2:** Results of orthogonal test.

No.	A	B	C	D	Extraction rate (%)
1	80	20	1.5	60	0.569
2	80	30	2	70	0.932
3	80	35	2.5	80	1.101
4	90	20	2	80	0.829
5	90	30	2.5	60	0.907
6	90	35	1.5	70	1.036
7	95	20	2.5	70	0.803
8	95	30	1.5	80	0.894
9	95	35	2	60	0.997
*K_1_*	0.867	0.734	0.833	0.824	
*K_2_*	0.924	0.911	0.919	0.924	
*K_3_*	0.898	1.045	0.937	0.941	
*R*	0.057	0.311	0.104	0.117	
*SS*	0.005	0.146	0.019	0.024	

**Table 3:** ANOVA results.

Variance source	Sum of squares of deviations	Degree of freedom	Variance	F	P
A	0.005	2	0.0025	1.00	
B	0.146	2	0.073	29.20	<0.05
C	0.019	2	0.0095	3.80	
D	0.024	2	0.012	4.80	
Error	0.005	2	0.0025		

Notes: F_0.01_(2,2) = 99.00; F_0.05_(2,2) = 19.00.

**Table 4:** Results of histopathologic observation.

Time (weeks)	Model Group (n * = 20)						Treatment Group (n = 20)						Blank Group (n = 4)
	Normal	Inflammation	Paraplasm			Squamous cell carcinoma	Normal	Inflammation	Paraplasm			Squamous cell carcinoma	Normal
			Mild	Moderate	Severe				Mild	Moderate	Severe		
3	19	1	0	0	0	0	20	0	0	0	0	0	4
6	1	10	4	4	1	0	5	10	3	1	1	0	4
9	1	2	12	1	3	1	4	6	4	5	1	0	4
12	0	0	5	4	4	7	0	5	4	4	3	4	4
Total	21	13	21	9	8	8	29	21	11	10	5	4	16

n = number of samples. Each roman number represent the number of samples.

**Table 5:** Different histologic incidences in model and treatment groups.

State	Model Group	Treatment Group
Normal	21	29
Inflammation	13	21
Paraplasm	38	26
Squamous cell carcinoma	8	4

Each roman number represent the number of samples.

**Table 6:** VEGF positive expression rates in model and treatment groups.

State	Model Group				Treatment Group			
	Grade 0	Grade 1	Grade 2	Positive rate (%)	Grade 0	Grade 1	Grade 2	Positive rate (%)
Normal	8	0	0	0	18	0	0	0
Inflammation	9	6	4	52.6	17	10	2	41.4
Paraplasm	9	10	26	80.0	14	11	4	51.7
Squamous cell carcinoma	1	2	5	87.5	1	2	1	75

Comparison of rates of two samples for intra-group comparison, and rank-sum test for inter-group comparison. Each roman number represent the number of samples.

**Table 7:** Caspase-3 positive expression rates in model and treatment groups.

State	Model Group				Treatment Group			
	Grade 0	Grade 1	Grade 2	Positive Rate (%)	Grade 0	Grade 1	Grade 2	Positive Rate (%)
Normal	4	4	0	50	13	5	0	27.8
Inflammation	8	7	4	57.9	15	10	4	48.3
Paraplasm	11	11	23	75.6	14	10	5	51.7
Squamous cell carcinoma	1	1	6	87.5	2	1	1	50

Comparison of rates of two samples for intra-group comparison, and rank-sum test for inter-group comparison. Each roman number represent the number of samples.

**Table 8:** Group allocation and disposition of test animals.

Group	Number	Disposition
Blank	8	After 3 weeks of breeding, apply normal saline to bilateral cheek membranes, and give 0.5% sodium carboxymethyl cellulose. 2 animals each are sacrificed at weeks 3, 6, 9 and 12.
Model	40	After 3 weeks of breeding, apply DMBA to bilateral cheek membranes, and give 0.5% sodium carboxymethyl cellulose. 10 animals each are sacrificed at weeks 3, 6, 9 and 12.
Treatment	40	After 3 weeks of breeding, apply DMBA to bilateral cheek membranes, and give ganoderma triterpenes drug liquid. 10 animals each are sacrificed at weeks 3, 6, 9 and 12.
